# Coronavirus disease 2019 on routine testing in eclampsia: a case report 

**DOI:** 10.1186/s13256-022-03308-8

**Published:** 2022-03-01

**Authors:** Pradip Kalsar, Shreya Datta, Arbabasu Kalsar, Andrew Marvin Kanyike

**Affiliations:** 1 Department of Gynecology, Mal Superspeciality Hospital, Mal, West Bengal India; 2 Department of Gynecology, Calcutta National Medical College, Kolkata, India; 3 Department of Obstetrics and Gynecology, Faculty of Health Sciences, Busitema University, Mbale, Uganda

**Keywords:** Eclampsia, Preeclampsia, COVID-19, Routine testing, Pregnancy

## Abstract

**Background:**

Coronavirus disease 2019 has been associated with adverse pregnancy outcomes, including preeclampsia. Coronavirus disease 2019 and preeclampsia have overlapping clinical features and are therefore challenging to differentiate. Since pregnant women are not routinely tested for coronavirus disease 2019, it is prudent to test for it among patients presenting with preeclampsia or eclampsia.

**Case presentation:**

A 23-year-old female, a Munda, gravida 1 para 0, at 36 weeks and 5 days of amenorrhea presented to Mal Super Specialty Hospital as a referral in a semiconscious state after a severe attack of tonic–clonic seizures. Detailed history from the husband was insignificant except for a persistent cough for the last 7 days. She had denied any visual changes, headaches, or vaginal discharge. Physical examination revealed tachycardia (150 beats per minute), elevated blood pressure (187/111 mmHg), tachypnea (36 breaths per minute), and oxygen saturation of 94% on room air. Routine coronavirus disease 2019 rapid test was positive, and urine dipstick was +3. Additional tests revealed leukocytosis and elevated liver enzymes. Chest radiograph revealed prominent interstitial markings, and a bedside transabdominal ultrasonography showed a live single intrauterine fetus in cephalic presentation with normal cardiac activity and movements. A diagnosis of a prime gravida with eclampsia and coronavirus disease 2019 was made. She was managed with intravenous labetalol; she had already received a loading dose of intravenous magnesium sulfate, and we administered two maintenance doses during monitoring. Within an hour of admission, she had a spontaneous rupture of the amniotic membranes, with meconium-stained liquor (grade 2), and the fetal heart rate (148 beats per minute) was reassuring. She had an uncomplicated vaginal delivery of a live male newborn. Shortly after delivery, she developed slight respiratory distress and significant fluid overload that was managed with furosemide. Coronavirus disease 2019 reverse-transcription polymerase chain reaction test came back negative for the neonate and positive for the mother. She was shifted to the coronavirus disease 2019 treatment unit, and her contact with the child was limited. She was kept on a course of tablets ivermectin, zinc, vitamin C, montelukast, azithromycin, metronidazole, and injectable pantoprazole. The mother and child were discharged on day 15 after recovery with negative COVID nasopharyngeal swab.

**Conclusion:**

A diagnosis of preeclampsia or eclampsia should prompt testing for coronavirus disease 2019.

## Background

Coronavirus disease 2019 (COVID-19), an infection caused by severe acute respiratory syndrome coronavirus 2 (SARS-CoV-2), was initially not attributed to serious maternal or neonatal morbidities [1, 2]. However, with the evolving literature, it has been reported to increase the risk for adverse pregnancy outcomes, causing higher rates of preterm birth, preeclampsia, cesarean delivery, and perinatal death especially among women with severe disease [3, 4]. COVID-19 is primarily a respiratory infection that, like preeclampsia in pregnancy, causes significant vascular changes through direct endothelial damage leading to hypertension and renal diseases, and affecting multiple organs [5]. The overlap in clinical features of COVID-19 and preeclampsia presents a challenge in differentiating them [6]. Mendoza and colleagues coined the concept of preeclampsia-like syndrome associated with severe COVID-19, which could be distinguished from actual preeclampsia by raised soluble fms-like tyrosine kinase-1/placental growth factor (sFlt-1/PlGF) ratio, lactate dehydrogenase (LDH), and uterine artery pulsatility index (UtAPI) [6], although these tests may not be readily available in many hospital settings. However, Papageorghiou *et al*. demonstrate that vascular changes that occur in preeclampsia, like in essential hypertension, predispose to susceptibility to contracting COVID-19 [4]. The interplay between the two conditions remains an area of interest for further research. Therefore, as we improve our understanding of the relationship, precautions for hypertensive disorders of pregnancy such as preeclampsia should be undertaken while monitoring pregnant women with COVID-19 infection [7]. Furthermore, although COVID-19 is not routinely tested for among pregnant women in most countries, it is prudent to rule it out among differentials in women presenting with preeclampsia–eclampsia clinical spectrum. In this case study, we present the case of a primigravida who was referred to our hospital with features of eclampsia and turned out to be COVID-19 positive.

## Case presentation

**Fig. 1:**
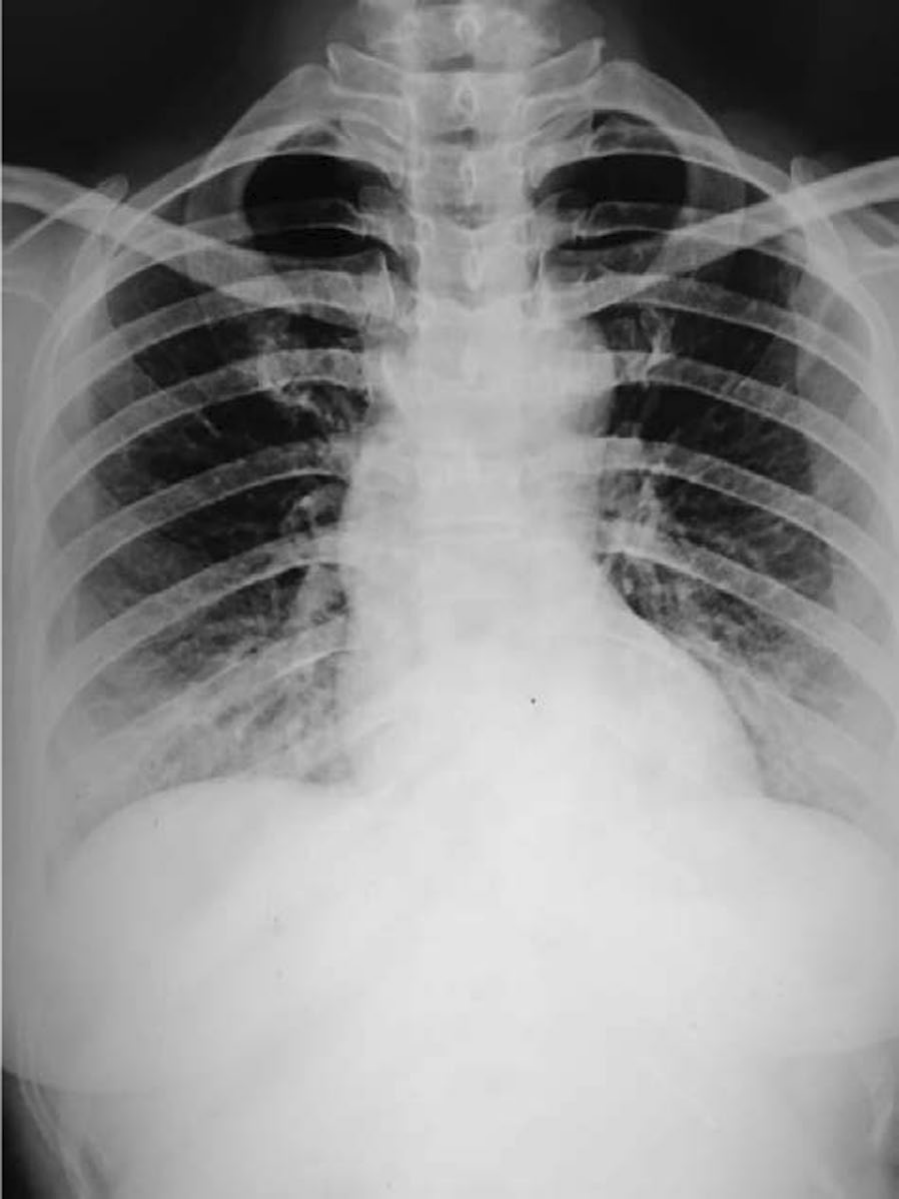
Chest X-ray showing prominent interstitial markings

A 23-year-old female, a Munda, gravida 1 para 0, at 36 weeks and 5 days of amenorrhea presented to the emergency department of Mal Super Specialty Hospital on 2 June 2021 as a referral from another facility in a semiconscious state due to a severe attack of tonic–clonic seizures. A detailed history taken from the husband was insignificant except for a persistent cough for the last 7 days. She denied any visual changes, headaches, or vaginal discharge. In the emergency department, her physical examination revealed tachycardia of 150 beats per minute, elevated blood pressure of 187/111 mmHg, tachypnea of 36 breaths per minute, and oxygen saturation of 94% on room air. Routine COVID-19 rapid antigen test (RAT) was positive, and urine dipstick was +3. Her blood was sent for additional laboratory tests (Table 1) and a chest X-ray was ordered (Fig. 1). A diagnosis of COVID-19 in eclampsia was made.

**Table 1:** Laboratory tests done during admission and at discharge of the patient

Blood investigation	Normal range	At the beginning of and during treatment	On discharge
Hemoglobin	12.1–15.1 g/dL	9.9 g/dL	9.1 g/dL
Urea	5–20 mg/dL	55 mg/dL	42 mg/dL
Creatinine	0.6–1.2 mg/dL	0.9 mg/dL	1.0 mg/dL
Serum sodium	135–145 mEq/L	139 mEq/L	136 mEq/L
Serum potassium	3.5–5 mEq/L	3.8 mEq/L	3.7 mEq/L
Serum calcium	2–2.25 mmol/L	1.15 mmol/L	1.21 mmol/L
C-reactive protein	1.5–27 mg/L	18.6 mg/L	7.4 mg/L
SGOT	8–45 units/L	276 units/L	121 units/L
SGPT	7–56 units/L	218 units/L	148 units/L
Alkaline phosphatase	44–147 IU/L	243 IU/L	112 IU/L
WBC count	6000–17,000/μL	21,200/μL	9700/μL

SGOT: Serum Glutamic-Oxaloacetic Transaminase, SGPT: Serum Glutamic Pyruvic Transaminase, WBC: White Blood Cell

Chest radiography images revealed prominent interstitial markings, and a bedside transabdominal ultrasonography revealed a live single intrauterine fetus in cephalic presentation with normal cardiac activity and movements.

On admission, she was promptly started on intravenous labetalol 20 mg that was repeated for one more dose after 15 minutes to control the blood pressure. She had been given a loading dose of intravenous magnesium sulfate (MgSO_4_) 4 g of 20% at 8:30 am from the referring facility for suspected eclampsia, and maintenance doses of 5 g of 50% MgSO_4_ were administered at 12:30 pm and 4:30 pm while monitoring for toxicity with patellar reflexes and respiratory rate prior to each dose at Mal Superspeciality Hospital. She was shifted to the critical care unit (CCU) and monitored. Within an hour of admission, she had a spontaneous rupture of the amniotic membranes, with meconium-stained liquor (grade 2); however, the fetal heart of 148 beats per minute was reassuring. The cervix dilated progressively up to 10 cm, leading to an uncomplicated vaginal delivery of a healthy, live-born male neonate. Shortly after delivery, she developed slight respiratory distress and her renal function (Table 1) worsened suddenly, resulting in significant volume overload and acute kidney injury. However, diuresis with intravenous furosemide 40 mg was administered on the evening of the delivery and followed up with the same dose after 12 hours, resulting in stabilization of her serum creatinine levels at 1.0 mg/dL as her hypoxic condition improved

COVID-19 reverse-transcription polymerase chain reaction (RT-PCR) came back negative for the neonate, while that of the mother returned positive the next day; she was shifted to the COVID-19 high-dependency unit (HDU). Due to her COVID-19-positive state, contact between mother and child was limited. During her entire stay at the COVID HDU, she was kept on a course of tablets (tab) ivermectin (once daily for 5 days),tab zinc (once a day), tab vitamin C (once daily), tab montelukast (once a day for 7 days), antibiotics ceftriaxone IV (for 5 days) and azithromycin 500 (for 7 days) each once daily, paracetamol 100 mg intravenously after delivery followed by tab paracetamol 650 three times per day, metronidazole 400 three times per day, and injection of pantoprazole 40 mg intravenously for 3 days followed by tab pantoprazole 40 mg orally twice daily. Mother and infant were discharged on day 15 after achieving complete recovery and a negative COVID-19 nasopharyngeal swab.

## Discussion

Eclampsia is defined as a seizure that occurs after 20 weeks gestation in the absence of epilepsy and other basic disorders. Eighty percent of eclampsia cases arise during the prenatal period and childbirth, although eclampsia has emerged in the postpartum period in a few cases. Even though preeclampsia (PE) and eclampsia do not always occur in succession, it was found that 79% cases of PE developed into eclampsia. It has been found that these patients can have symptoms of brain edema (visual symptoms, headache) even when blood pressure remains normal [8]. The exact mechanism of seizure in eclampsia is not clear, but is most likely secondary to a combination of cerebral edema, ischemia, and transitory vasospasm of the cerebral vasculature [9].

The co-existence of COVID-19 and preeclampsia or eclampsia synergistically increases the risks of adverse pregnancy outcomes such as preterm birth, severe perinatal morbidity and mortality, and adverse maternal outcomes [4]. During pregnancy, the angiotensin-converting enzyme 2 (ACE2) receptor that mediates SARS-CoV-2 actions in COVID-19 is abundantly expressed in the placental tissue and plays a vital role in the regulation of arterial pressure, which is disrupted, leading to vasoconstriction and preeclampsia [5, 10].

The relationship between COVID-19 and preeclampsia–eclampsia spectrum could be viewed from different angles; COVID-19 can manifest signs and symptoms that fulfill the diagnostic criteria for preeclampsia; alternatively, COVID-19 could be on an etiologic road toward preeclampsia, or preeclampsia could create a milieu that increases the risk of contracting COVID-19 [4]. Papageorghiou *et al*. from a longitudinal prospective study showed that COVID-19 in pregnancy is independently associated with preeclampsia especially among nulliparous women irrespective of severity of symptoms. Furthermore, they report that prevalence of COVID-19 was highest within 33–37 weeks of gestation when preeclampsia typically manifests clinically [4]. In our case study, the patient was nulliparous in the 36th week of gestation, which reinforces these findings.

Existing literature has indicated that there is little risk of vertical transmission of COVID-19 to the fetus [11]. In a case series of pregnant women with COVID-19, only one newborn tested positive for SARS-CoV-2 within the first 24 h of life using a nasopharyngeal swab test [12]. In our case report, the neonate tested negative for COVID-19 in accordance with most studies that have reported no vertical transmission of SARS-CoV-2.

At present, there are no definite guidelines for early detection or prevention of late-onset postpartum eclampsia in a patient without prior eclampsia [13]. Managing hypertension and preventing convulsions are key elements in treating severe preeclampsia/eclampsia. While any of several antihypertensive drugs may be used to treat severe preeclampsia/eclampsia, MgSO_4_ is the clear drug of choice to prevent convulsions, and its potential impact on maternal morbidity and mortality is considerable [14]. In Kano, Nigeria, for example, the case fatality rate for severe preeclampsia/eclampsia fell from 20.9% to 2.3% after MgSO_4_ was introduced; perinatal mortality also fell significantly [15]. Although there is a theoretical concern that treatment with magnesium sulfate could worsen SARS-CoV-2 infection given the possibility of its respiratory depression, Joudi and colleagues safely administered magnesium sulfate with an intravenous loading dose of 4 g and maintenance doses like in our case study without adversities [16]. This bolsters the conclusion by Boelig *et al*. that magnesium can be used as indicated in pregnant women with COVID-19 [17].

Both eclampsia and COVID-19 are examples of microvascular disease causing endothelial injury. They both cause a high prothrombotic tendency leading to multiorgan failure [10]. With the limited literature at hand, the obstetricians and other healthcare workers attending to pregnant women should be cognizant of their interplay and be vigilant to rule out COVID-19 among women presenting with preeclampsia–eclampsia clinical spectrum

## Conclusion

The clinical dilemma of differentiating COVID-19 from preeclampsia–eclampsia spectrum is currently being faced by clinicians during the pandemic. In the event of diagnosing one of the conditions, a high index of suspicion should prompt investigations for the other to avoid missed opportunities of such fatal conditions. Clinical data on the adverse effects of COVID-19 infection on pregnant women and the list of accompanying complications are severely deficient. Therefore, there is need for more research to characterize the interplay of COVID-19 within the physiologically deranged milieu of pregnancy.

## Data Availability

Data sharing is not applicable to this article as no datasets were generated or analyzed during the current case report

## Abbreviations

COVID-19: Coronavirus disease 2019
RT PCR: Reverse-transcription polymerase chain reaction
PlGF: Platelet growth factor
LDH: Lactate dehydrogenase
CCU: Critical care unit
HDU: High-dependency unit
